# Controversial Aspects in Sedative Techniques for Drug-Induced Sleep Endoscopy (DISE)—A Narrative Review

**DOI:** 10.3390/medsci14010058

**Published:** 2026-01-24

**Authors:** Narcis-Valentin Tănase, Catalina Voiosu, Luana-Maria Gherasie

**Affiliations:** 1 Department of Anaesthesia and Intensive Care Medicine, Carol Davila University of Medicine and Pharmacy, 050474 Bucharest, Romania; 2 Clinic of Anaesthesia and Intensive Care Medicine, Dr. Carol Davila Central University and Emergency Military Hospital, 010825 Bucharest, Romania; 3 Department of E.N.T., Carol Davila University of Medicine and Pharmacy, 050474 Bucharest, Romania; luana-maria.bujor@drd.umfcd.ro; 4 Institute of Phonoaudiology and Functional Surgery Prof. Dr. D. Hociotă, 061344 Bucharest, Romania

**Keywords:** drug-induced sleep endoscopy, obstructive sleep apnea, sedative procedure, TCI, general-purpose PK-PD models

## Abstract

**Background/Objectives:** Drug-induced sleep endoscopy (DISE) is used in obstructive sleep apnea (OSA) to visualize dynamic upper airway collapse, but sedation protocols vary widely with no consensus on the optimal agent or technique. This narrative review aims to clarify current sedation strategies for DISE in OSA and their clinical implications. **Methods:** We systematically searched PubMed, Scopus, Web of Science, and Cochrane Library for English-language publications on DISE sedation (2000–2025). Relevant clinical studies, guidelines, and reviews were included. Data were qualitatively synthesized due to heterogeneity among studies. **Results:** Sedation approaches in DISE varied considerably. Propofol, dexmedetomidine, and midazolam were the primary agents identified. Propofol provided rapid, titratable sedation but increased airway collapsibility at higher doses; dexmedetomidine produced a more natural sleep-like state with minimal respiratory depression; midazolam was less favored due to prolonged effects. Use of target-controlled infusion (TCI) and pharmacokinetic–pharmacodynamic (PK–PD) models improved control of propofol sedation. Co-sedative adjuncts (e.g., opioids) reduced the required sedative dose but added risk of respiratory depression. Careful titration to the lowest effective dose-often guided by bispectral index (BIS) monitoring—was emphasized to achieve adequate sedation without artifactual airway collapse. No universal DISE sedation protocol was identified. **Conclusions:** Optimal DISE sedation balances adequate depth with patient safety to ensure reliable findings. Using the minimum effective dose, guided by objective monitoring (e.g., BIS), is recommended. There is a need for standardized sedation protocols and further research (e.g., in obese patients) to resolve current controversies and improve DISE’s utility in OSA management.

## 1. Introduction

Obstructive sleep apnea (OSA) is among the most common sleep-disordered breathing disorders [1], with a reported global prevalence of approximately 22.6% in the general population [2]. A highly heterogeneous clinical presentation characterizes this pathological condition, and accurately diagnosing it, describing the OSA phenotype, and consequently tailoring an appropriate treatment are paramount. OSA induces serious chronic clinical consequences: obesity, increased cardiovascular risk, cognitive deterioration, arterial hypertension, and elevated risk of implication in vehicle accidents [3].

Drug-induced sleep endoscopy (DISE) was developed to visualize the upper airway during a pharmacologically induced “sleep” state, providing dynamic information complementary to polysomnography [4].

Consequently, the clinical assessment of the anatomy and dynamics of the obstruction site(s) of the respiratory airway during sleep remains critical to the successful surgical treatment of patients with OSA [1]. Moreover, a subset of these patients cannot tolerate nocturnal continuous positive pressure ventilation (CPAP) or refuse CPAP treatment.

Drug-induced sleep endoscopy (DISE) is an adjuvant diagnostic tool for evaluating patients with OSA and is complementary to polysomnographic data [5]. Its indications were delineated in previous Consensus position papers [5,6,7]. DISE offers upper airway (UA) data that are important for selecting the appropriate surgical approach, but the impact on surgical outcomes remains widely debated [8,9]. DISE remains useful and is recommended to avoid unnecessary surgeries or surgeries with very low success rates (as recommended by the Guidelines of the French Society of ENT) [10] and to evaluate patients with prior surgical failure in OSA [11]. A recent systematic review including 25 studies and 1522 patients reported a failure rate of up to 37% for DISE-guided upper airway surgery, indicating that outcomes remain variable [11]. Furthermore, DISE effectively identifies obstruction sites associated with surgical failure and aids in planning both single-level and multilevel surgical interventions [11]. The choice of sedative technique is essential to DISE’s accuracy and clinical utility, as it influences both the safety of the procedure and the reliability of diagnostic findings.

However, significant controversies persist regarding which sedative agents and protocols are optimal in replicating natural sleep, minimizing adverse events, and yielding reproducible, clinically meaningful results. These controversies are compounded by methodological variability, absence of standardized assessment tools, and patient-specific risk factors, making the optimization of DISE sedation a critical and unresolved issue in sleep medicine and anesthesiology [12,13].

Sedation technique is central to DISE’s success, as it must achieve a sleep-like state without compromising patient safety or distorting the airway findings. The chosen sedative agent and depth of sedation influence muscle tone in the pharyngeal dilators, airway reflexes, and breathing–all of which affect whether the endoscopic findings truly represent the patient’s natural sleep anatomy [4]. The ideal sedative for DISE would closely mimic natural sleep (particularly non-REM sleep) in terms of airway muscle activity and respiratory pattern, while allowing easy titration and rapid recovery [14]. In practice, however, there is significant variability in the medications and protocols used for DISE across institutions. Propofol (an intravenous hypnotic that potentiates GABA type A receptors), midazolam (a short-acting benzodiazepine that enhances GABAergic inhibitory neurotransmission), and dexmedetomidine (an α2-adrenergic agonist) are the most common agents, but no universally accepted regimen exists.

Each agent’s pharmacology affects upper airway physiology differently: for example, propofol causes dose-dependent depression of the genioglossus muscle and airway reflexes, whereas dexmedetomidine induces a more natural sleep-like sedation with minimal respiratory depression. Choosing the right sedative (or combination of sedatives) and administering it in a controlled manner is critical to achieve sufficient sedation without precipitating artifactual airway collapse.

This narrative review provides a contemporary overview of sedative techniques for DISE, focusing on the controversies and recent evidence. We first discuss the mechanistic effects of the key sedative agents on upper airway physiology and compare their performance in DISE. We then examine the use of co-sedatives, sedation delivery systems and models, monitoring of sedation depth, and special considerations such as protocol standardization and obese patients. Throughout, we distinguish well-established findings from emerging data or expert opinion. By clarifying what is known and what remains unknown, our goal is to support clinicians in making informed sedation decisions for DISE and to highlight priorities for future research to optimize this procedure.

## 2. Methods: Literature Research

This review was conducted and reported in alignment with the Scale for the Assessment of Narrative Review Articles (SANRA) guidelines to ensure methodological rigor and transparency.

We performed a comprehensive literature search of PubMed, Scopus, Web of Science, and the Cochrane Library for publications from 2000 through November 2025. The primary search terms were based on the following Boolean string: (“drug-induced sleep endoscopy” OR “DISE”) AND (“sedation” OR “sedative technique”) AND (“propofol” OR “dexmedetodine” OR “midazolam”) AND (“target-controlled infusion” OR “TCI” OR “pharmacokinetic-pharmacodynamic model” OR “PK-PD model”) AND (“obstructive sleep apnea” OR “OSA”). The search was limited to English-language publications. Reference lists of relevant articles were manually screened to identify additional eligible studies.

Inclusion criteria: clinical guidelines and consensus papers; proposed protocols; comparative cohort studies; randomized controlled trials; systematic reviews; meta-analyses; and significant narrative reviews related to DISE sedation. Both adult and pediatric studies were considered if relevant. Key sources such as the 2017 European position paper on DISE and recent meta-analyses were prioritized to capture prevailing expert opinions and evidence. Exclusion criteria: we excluded small case series, conference abstracts, and studies focusing on sedative drugs outside the context of DISE or OSA.

Given the heterogeneity of study designs and the lack of randomized trials in certain aspects of DISE sedation, a narrative review was deemed more appropriate than a formal systematic review. This approach allowed us to integrate findings from diverse study types (including expert consensus and physiologic studies) and to discuss conceptual controversies in depth. We did not perform quantitative meta-analysis of outcomes due to variability in reported endpoints across studies. Instead, we qualitatively synthesized the evidence, highlighting points of agreement, disagreement, and knowledge gaps. The SANRA checklist was used as a framework to ensure that our literature search, selection, and synthesis processes were well documented and that bias was minimized.

## 3. Main Controversies

### 3.1. Sedative Agent Selection: Propofol, Dexmedetomidine, or Midazolam?

The current clinical perspective on approaching sedative strategy registers a large variability among clinicians [6]. Differences emerge concerning the ideal selection of drugs, sedative protocols, the efficacy of combination strategies, and the precision of dosing.

The most commonly used drugs for DISE are propofol, midazolam, and dexmedetomidine [15,16]. Each of these agents exhibits distinct pharmacokinetic and pharmacodynamic characteristics that influence onset of action, titrability, recovery profile, and upper airway behavior during DISE.

Propofol, the most studied sedative agent in this context, exhibits certain similarities to the UA physiology encountered during the transition from wakefulness to unconsciousness in natural sleep [12,13,17]. Despite this intrinsic advantage, performing DISE at the lowest effect-site propofol concentration could offer significant benefits in terms of minimizing the risk of pharmacologically induced airway collapse [18]. However, using propofol for DISE sedation may also carry a higher risk of airway collapsibility with increasing effect-site concentrations [4].

From a pharmacokinetic perspective, propofol is characterized by rapid distribution and clearance, allowing fine titration—particularly when administered via target-controlled infusion—while its pharmacodynamic effects on upper airway muscle tone are strongly dose-dependent.

The propofol sedative technique for DISE can be performed using three methods: iterative bolus technique, manually controlled infusion (MCI), and target-controlled infusion (TCI). In manually controlled infusion, a dosage is administered at a rate of 50–100 mL/h, with adjustments made based on the patient’s clinical response. The bolus technique involves various administration protocols based on clinician preference, starting with an initial dose of 30–50 mg, followed by repeated doses of 10 mg every 2 min, or starting at 1 mg/kg and subsequently administering 20 mg every 2 min [6]. De Vito et al. [19] conducted a comparative study between bolus and continuous infusion techniques, revealing that the bolus method resulted in more significant oxygen desaturation. The bolus group from this study experienced an abrupt pharyngeal collapse without accompanying snoring or apneic events, and the authors affirmed a rapid pharmacological effect rather than a gradual natural muscle relaxation; this abrupt sleep induction bypassed the diagnostic window during which pharyngeal collapse is most likely to happen, thereby affecting the diagnosis accuracy. Concerning the use of propofol compared to dexmedetomidine in sedation practice for DISE, some authors emphasized the significantly different effect on the pattern of upper airway obstruction observed during DISE, raising concerns about optimal pharmacologic protocol and sedative technique for DISE [14,16,20].

Propofol offers rapid induction and a short recovery, enabling fine titration of sedation and a rapid wake-up after the procedure. Mechanistically, propofol causes dose-dependent relaxation of upper airway dilator muscles and suppression of protective reflexes. As propofol effect-site concentration increases, the critical closing pressure (P_crit_) of the pharynx rises (becomes less negative), indicating a more collapsible airway. Eastwood et al. [4] demonstrated that increasing propofol concentrations (from ~2.5 to 6.0 μg/mL at the effect site) progressively elevated P_crit_ and reduced genioglossus muscle activity, resulting in significantly greater airway collapsibility. Thus, while propofol can successfully induce a sleep-like state, overshoot in propofol depth can precipitate collapse that would not occur in natural sleep, a key concern for diagnostic accuracy. On the other hand, when carefully titrated to a moderate level, propofol sedation yields collapse patterns that are generally comparable to those of natural non-REM sleep. A study [21] using simultaneous polysomnography found that propofol-induced DISE typically achieved Stage N_2_ sleep and reproduced obstruction sites similar to those during natural sleep in most airway regions. Notably, propofol tends to cause more relaxation at the velum (soft palate), sometimes producing complete velopharyngeal collapse more readily than midazolam. Overall, propofol’s clinical advantages include its familiarity, controllability (especially with infusion systems), and widespread availability. Its drawbacks include dose-dependent respiratory depression, hypotension, and the risk of inducing non-physiologic levels of airway flaccidity if not carefully titrated. Indeed, performing DISE at the lowest effective propofol concentration is recommended to minimize these risks [22].

Dexmedetomidine (DEX) is a selective α2-adrenergic agonist that produces a unique form of sedation often similarized to natural sleep. Unlike propofol, dexmedetomidine provides sedation with analgesia and minimal respiratory depression, primarily by action on locus coeruleus neurons and endogenous sleep pathways.

Dexmedetomidine exhibits a distinct pharmacokinetic profile, with a slower onset and longer context-sensitive half-time compared with propofol, which limits rapid titration but provides stable sedation once achieved. Pharmacodynamically, its α2-agonist mechanism preserves respiratory drive and upper airway muscle tone, resulting in less pronounced airway collapsibility at comparable sedation depths.

Patients sedated with dexmedetomidine are often arousable and maintain relatively stable ventilatory drive. In the context of DISE, dexmedetomidine’s sedation is closer to physiologic sleep in terms of EEG patterns (resembling stage II non-REM). It preserves upper airway muscle tone better than GABAergic drugs [23]. Clinically, this means less complete airway collapse is observed under dexmedetomidine, which can be a double-edged sword. On the one hand, dexmedetomidine tends to maintain higher minimum oxygen saturation and fewer apnea/hypopnea events during DISE than propofol. For example, Yoon et al. [14] reported that OSA patients had significantly higher SpO_2_ nadirs and fewer deep desaturations with dexmedetomidine; none dropped below 80% O_2_ with DEX, whereas several did with propofol. Dexmedetomidine also provided greater hemodynamic stability, with fewer patients experiencing >20% blood pressure or heart rate changes. These traits underscore dexmedetomidine’s safety advantages for high-risk patients (e.g., severe OSA or cardiovascular comorbidities). On the other hand, because DEX does not relax the airway to the same extent, it may underrepresent the worst-case collapse that some OSA patients experience during REM sleep or deeper anesthesia. In fact, when the depth of sedation is comparable, studies have found that the patterns of upper airway collapse are qualitatively similar with dexmedetomidine and propofol–all obstruction sites seen with propofol were also seen with DEX in the same patients, according to Yoon et al. [14]. However, the degree of narrowing at some sites was slightly less with dexmedetomidine. This suggests that DEX can adequately reveal the presence and location of obstructions, but might not fully reproduce the severity of collapse that propofol can elicit (which could correlate to REM sleep events). Another consideration is dexmedetomidine’s pharmacokinetics: it has a relatively slow onset (5–10 min to effect) and longer-lasting sedative effects after infusion off, which can prolong recovery. During DISE, this slower titration can be a disadvantage when trying to rapidly achieve the desired sedation level or if quick adjustments are needed. DEX also commonly causes bradycardia and mild hypotension due to central sympatholysis, though usually without severe consequences if monitored. In summary, dexmedetomidine offers a more “natural” sleep mimicry and a high safety margin for breathing, at the cost of slower control and a possibility of missing the most severe collapse that might occur in real sleep [24].

Midazolam is a benzodiazepine sedative occasionally used in DISE, particularly in centers or situations where propofol or DEX are less accessible. Midazolam has a slower onset than propofol (peak effect in ~2–3 min) and a longer, less predictable duration (due to active metabolites), making titration more challenging. Like propofol, midazolam is a GABA type A receptor agonist and causes central nervous system depression, muscle relaxation, and anterograde amnesia.

Midazolam’s pharmacokinetic profile is characterized by hepatic metabolism with active metabolites and variable clearance, which contributes to less predictable recovery and accumulation during prolonged or repeated dosing; pharmacodynamically, its GABAergic effects reduce upper airway muscle tone in a dose-dependent manner.

Comparative studies of midazolam vs. propofol in DISE showed that midazolam can achieve similar obstruction findings if carefully dosed. For instance, Carrasco-Llatas et al. [21] sedated the same patients with propofol on one day and midazolam on another; both sedatives produced comparable Stage N_2_ sleep states and showed good agreement in collapse patterns across most airway levels. Notably, midazolam and propofol had equivalent effects on upper airway patency at moderate sedation, with similar pharyngeal P crit values (~−5 cmH_2_O)–on the order of what is seen in natural sleep. This suggests that midazolam, when titrated to moderate sedation, maintains tonic neuromuscular activity of the airway dilators comparable to that of propofol and to normal sleep. However, midazolam’s disadvantages include a greater tendency for respiratory depression (especially when combined with opioids), no easy method for continuous infusion control in standard practice, and prolonged sedation or grogginess post-procedure due to its longer half-life. Clinicians have also noted less fine control over sedation depth with midazolam. In DISE, midazolam has been associated with a higher incidence of significant oxygen desaturation events than propofol in some studies, likely because its effects can accumulate and exceed target limits before adjustments are made. On the positive side, midazolam is widely available, familiar to many providers, and can be reversed with flumazenil if needed—a safety feature not available for propofol or dexmedetomidine. In practice, midazolam is often used in combination with other agents or for lighter sedation protocols [21]. Its use as a sole agent for DISE has decreased as TCI propofol and dexmedetomidine techniques have advanced, but it remains an option in settings where simplicity and availability are priorities. Recently, remimazolam has shown its feasibility and a superior safety profile compared to propofol [25].

In summary, each sedative agent offers a different balance of airway effects, onset/recovery profile, and reliability of mimicking sleep (Table 1). Propofol is fast and controllable but requires vigilance to avoid excessive collapse; dexmedetomidine preserves breathing and simulates natural sleep well but is slower and can underplay worst-case collapse; midazolam is feasible and similar to propofol in moderate sedation effects, but is less titratable and can unpredictably depress respiration. The selection often depends on clinician preference, patient factors, and resource availability. Importantly, emerging evidence [6] suggests that when sedation is maintained within a moderate range (e.g., target effect-site concentrations for propofol of ~2–3 µg/mL, or an equivalent depth for midazolam or DEX), all three agents can provide a reasonable simulation of non-REM sleep in patients with OSA. More profound sedation with any agent, however, will increase airway collapsibility and risk compromising the diagnostic value of DISE. Thus, regardless of agent choice, achieving the appropriate depth of sedation is paramount.

These pharmacokinetic and pharmacodynamic differences among sedative agents underscore the need for individualized drug selection and careful titration during DISE to balance diagnostic fidelity with patient safety.

### 3.2. Co-Sedatives: Utility and Risks

Regarding the addition of co-sedatives (e.g., opioids) to the sedative regimen for drug-induced sleep endoscopy (DISE), there are both advantages and disadvantages. On the one hand, opioids can enhance procedural tolerance and sedation [27,28,29], providing a more comfortable experience for patients and potentially improving airway visualization. However, on the other hand, the use of opioids introduces significant risks, such as respiratory depression and an increased likelihood of sedation-related complications [28]. Remifentanil TCI, in addition to a sedative regimen based on propofol TCI, can reduce the propofol effect-site concentration required for airway endoscopy [27,29]. The optimal remifentanil concentration with respect to the risk-benefit balance in the effect compartment was 1.0 ng/mL in the Minto model, when combined with propofol administered according to the Schnider PK-PD model for DISE [27].

### 3.3. Sedation Protocols-Target-Controlled Infusion Devices

The European consensus update from 2017 [6] and the Guidelines of the French Society of ENT [10] recommend TCI as the preferred method for administering propofol sedation in DISE, intending to accurately reproduce respiratory events occurring in natural sleep, with an initial effect-site concentration target between 2.0 and 2.5 µg/mL, subsequently increasing the dose by 0.2–0.5 µg/mL until the desired sedation level is achieved.

Target-controlled infusion systems deliver sedative drugs via software-managed infusion pumps to achieve a desired target concentration in the blood or the brain’s effect compartment. This method allows for rapid attainment of the target concentration and maintenance of the desired effect through integrated software algorithms [30]. Numerous studies have demonstrated the advantages of TCI technology for administering propofol sedation during DISE, making it the recommended standard of care [5,6,13,17,19]. TCI sedation for DISE is marked by a reduced incidence of adverse events (i.e., hypotension, bradycardia, desaturation) than bolus administration [19].

The primary TCI models used are those developed by Marsh [31] and Schnider [32]. The Schnider model is a well-established pharmacokinetic model widely used in clinical settings, extending the Marsh model for TCI systems. Recently, Eleveld introduced a new pharmacokinetic-pharmacodynamic (PK-PD) model for propofol designed to accommodate a diverse population range [33]. Its general-purpose nature offers several advantages, including minimizing the risk of incorrectly selecting a specific model for an individual patient, reducing the need for extrapolation, and overcoming the potential unfamiliarity that clinician anesthesiologists may have with existing models. This model aims to address limitations found in earlier models, improving the accuracy of drug concentration predictions and minimizing the risk of selecting an inappropriate model for individual patients, as well as altering resulting concentrations based on clinician familiarity [34]. The Eleveld model exhibited a shorter time to reach the optimal sedation level, reduced overall procedural duration, and a notable difference in effect-site concentration at the end of the endoscopy when compared to the Schnider model. The incidence of adverse events was similar between both groups, indicating that the Eleveld model may enhance efficiency while maintaining safety during DISE [35].

Regarding the optimal selection of a pharmacokinetic-pharmacodynamic (PK-PD) model for propofol sedation delivery in DISE, the literature provides little scientific data, and the Schnider model is likely the most commonly used PK-PD model in this context. There is a relative lack of studies comparing various pharmacokinetic-pharmacodynamic (PK-PD) models in TCI propofol sedation for DISE concerning the efficiency, side effects, and feasibility. Although TCI systems provide more accurate sedation depth control in DISE, clinicians must exercise caution when transitioning from established models (such as Schnider and Marsh) to general-purpose models (such as Eleveld), as targeting the same concentrations in the effect compartment may expose the patient to overdose and adverse airway events such as respiratory depression and unintended results during DISE [35].

**Figure 1:**
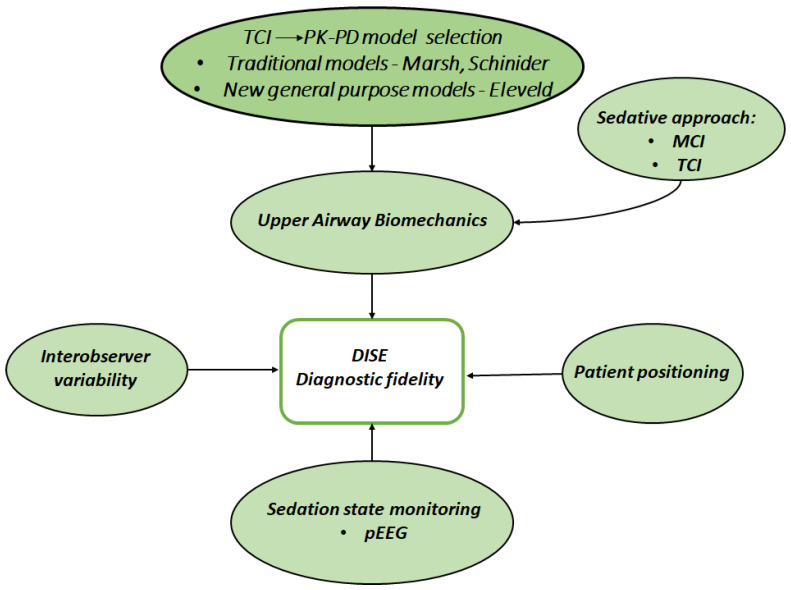
Conceptual framework of PK–PD–guided sedation in DISE. Selection of the PK–PD (pharmacokinetic-pharmacodynamic) model for target-controlled infusion (TCI)—including traditional models (e.g., Marsh, Schnider) and newer general-purpose models (e.g., Eleveld)—determines effect-site targeting and sedation depth. Together with the sedative approach (manually controlled infusion, MCI, or TCI), sedation state monitoring using processed electroencephalography (pEEG), patient positioning, and inter-observer variability, these factors modulate upper airway biomechanics and ultimately influence the diagnostic fidelity of drug-induced sleep endoscopy (DISE).

Eastwood et al. [4] demonstrated a dose-dependent increase in airway collapsibility with increasing propofol dose in DISE; consequently, performing endoscopy at the lowest clinically effective effect-site concentration of propofol offered benefits in terms of diagnostic consistency. Figure 1 seeks to integrate all the elements that can intricatelly contribute to achieving an accurate diagnosis using DISE.

### 3.4. Combination of Sedation Regimens

Several combination sedative regimens are proposed in the literature, each of them presenting some advantages and undesirable side effects. Combining dexmedetomidine with propofol or ketamine can optimize sedation onset, recovery times, and airway safety, especially in pediatric and high-risk patients [36,37,38,39]. In a systematic review [40], Eshan et al. found a notable lack of literature on the effects of combining anesthetic agents on the upper airway, which is critical given that most DISE protocols involve combinations of agents (e.g., dexmedetomidine and ketamine). Most studies indicated that inhalational anesthetics and opioids exacerbated dynamic airway collapse, suggesting they are not ideal choices for this procedure. Additionally, local anesthetics, typically used to reduce the risk of laryngospasm, may influence airway reflexes; therefore, they should be used only if the intention is to traverse the vocal cords at the end of a DISE procedure [6].

### 3.5. Monitoring Sedation Depth

The ideal depth of sedation required for effective visualization during endoscopy, without compromising patient safety or yielding false-positive results (e.g., obstructions induced by pharmacological effect on the upper airway rather than reproduced sleep), remains controversial. Deeper sedation increases airway collapsibility, affecting diagnosis accuracy. Some authors recommend bispectral index (BIS) monitoring with a target BIS of 65–75 [41]. BIS monitoring cannot discriminate among sleep stages [42] and is not helpful during sedation with dexmedetomidine or ketamine [43].

DISE is not helpful for patients who exhibit apnea events in paradoxical (REM) sleep [10], because sedative drugs do not have the potential “to imitate” this sleep pattern.

Heiser et al. [18] demonstrated in a recent study that during DISE with propofol TCI, upper airway collapse was observed at moderate levels of sedation. Consequently, surgical treatment decisions could be made based on observations at this sedation level. Further increases in sedation did not enhance treatment outcomes and should be avoided to ensure the safety of DISE.

To determine the appropriate level of moderate sedation, employing a sedation depth-monitoring method such as entropy or BIS is advantageous. Objective monitoring of sedation depth in DISE has predominantly been studied using BIS so far. More contemporary pEEG-based monitoring options could offer advantages in more precisely quantifying sedation depth and, in clinical practice, achieving the optimal level required for accurate airway assessment [23,44].

Notably, there is no single accepted BIS range for DISE; different groups have recommended targets from approximately 50 up to 80 [5,6]. For instance, Lo et al. suggested that maintaining BIS ~ 65–75 yields optimal DISE conditions [41,43]. This variability reflects the lack of consensus on sedation depth, underscoring that DISE sedation must be individualized to achieve sleep-like collapse without over-sedation. Monitoring the depth of sedation during DISE is also sometimes challenging due to the discordance among various EEG-derived indices, as evidenced by the observation that two-thirds of cases generated conflicting recommendations despite identical EEG signals [45].

**Figure 2:**
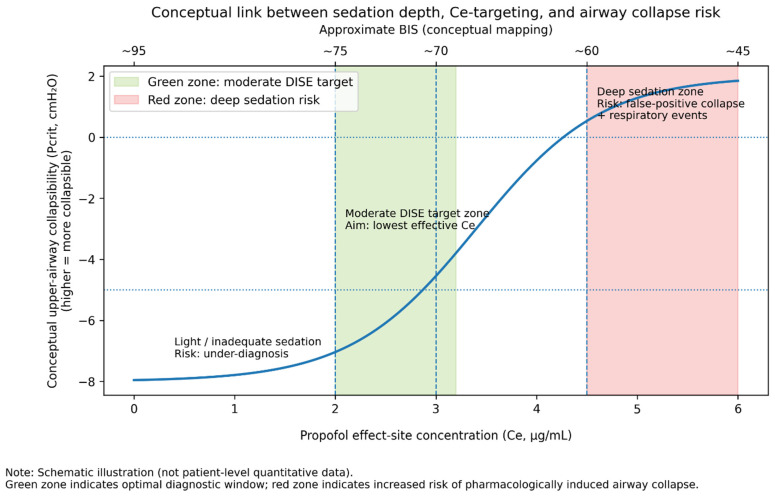
Sedation depth, drug concentration, and airway collapse risk. At moderate sedation (green zone), the effect-site concentration (Schnider model) of propofol is around 2–3 µg/mL, and the patient maintains partial upper airway muscle tone, allowing observation of obstructions comparable to natural sleep. As sedation deepens (propofol > 4–5 µg/mL effect-site, BIS < 60), airway collapse risk increases sharply due to loss of muscle tone. The critical closing pressure (P crit) becomes less negative (moves toward zero or positive), indicating a collapsible airway. Beyond this threshold (red zone), further depth adds no diagnostic value and may produce false collapse. Sedation depth should thus be titrated into the range that just produces the patient’s natural collapses and no more. (Data for propofol effect-site vs. P crit from Eastwood et al., Ref. [4]).

In conclusion, monitoring sedation depth in DISE is advantageous to balance safety and diagnostic accuracy. BIS is the most studied tool; it should be used with an understanding of its limitations. Based on international consensus recommendations on DISE, the optimal observational window corresponds to moderate sedation levels, typically within a BIS range of approximately 60–80 when propofol is used [6]. Future advances in sedation monitoring, potentially tailored to sleep endoscopy (e.g., by detecting when “sedative sleep” approximates natural sleep EEG), could further improve the procedure. Figure 2 illustrates the conceptual relationship among sedation depth, drug concentration, and airway collapse risk, emphasizing that beyond a certain depth, the risk of iatrogenic airway closure rises steeply.

### 3.6. Standardization of Protocols

Evidence indicates that propofol sedation impacts neural pathways not involved in natural sleep, leading to alterations in sleep architecture compared with typical sleep [13]. Additionally, propofol causes dose-dependent increases in airway collapsibility and reductions in genioglossus muscle tone [13]. These factors raise concerns that sleep endoscopy utilizing propofol requires careful titration to prevent false-positive results due to artificially induced airway collapse, as noted in the senior author’s clinical experience. This characteristic of the drug underscores the importance of administering the lowest effective dose of propofol, ideally through a targeted control infusion (TCI) system.

One of the overarching issues in DISE is the lack of a standardized sedation protocol across different practitioners and institutions. This variability spans the choice of drugs, dosing methods, use of adjuncts, patient positioning, and scoring of findings. Such heterogeneity can affect both the immediate findings of the DISE exam and the long-term surgical outcomes based on those findings.

The latest European position paper on DISE [6] explicitly identified standardization of the DISE technique as a key research priority. Despite this, little progress has been made in harmonizing practices internationally. Surveys indicate that some centers use propofol TCI with BIS monitoring, others use bolus midazolam, and yet others use dexmedetomidine infusions–all with differing criteria for what constitutes adequate sedation. This makes it difficult to compare studies or pool data; an outcome of “DISE-positive for tongue collapse” could mean different things if one site’s patient was deeply sedated with propofol vs. another site using a lighter DEX sedation.

Why does standardization matter? Because the sedation method can influence airway dynamics, it could ultimately influence clinical decisions (e.g., whether to operate and on which level). Sedation depth, for example, significantly affects airway collapsibility and observed obstruction patterns. Traxdorf et al. [17] showed that deeper propofol levels increased multilevel collapse, whereas lighter sedation might show only partial collapse. If one clinician always sedates deeply, they might label a patient as having multilevel (e.g., palate + tongue + epiglottis) obstruction and opt for multi-level surgery, whereas another clinician using moderate sedation might see only palatal collapse and suggest a single-level surgery. Both could be “right” under their conditions, but it leads to inconsistent care.

Inter-observer variability in DISE interpretation further compounds the issue–studies reported only moderate agreement between different surgeons scoring the same video, particularly for subtle airway levels [46]. Velum and oropharynx findings tended to have better agreement, whereas hypopharynx (tongue base, epiglottis) often had poor reliability. Part of this variability could be due to actual differences in sedation conditions at the time of observation. It’s plausible that establishing a uniform sedation protocol (drug, target depth, etc.) would reduce one source of variability, allowing more focus on true anatomical differences [47]. From a surgical outcome perspective, the inconsistent use of DISE and varied sedation approaches have made it challenging to demonstrate DISE’s value in improving OSA surgery outcomes. Some recent evidence has raised questions: A recent randomized trial and a systematic review found that performing preoperative DISE did not significantly improve surgical success rates compared to surgery without DISE [48,49,50] and concluded that in primary OSA cases, a pragmatic multilevel surgery without DISE was as effective as DISE-guided surgery in terms of outcomes, suggesting that routine DISE might not always change the game. However, these findings come with caveats–if DISE is done inconsistently or interpreted variably, its benefit could be masked. A counterpoint is a 2017 study by De Vito et al. [19] that showed better surgical outcomes in patients who underwent DISE with a refined sedation technique (propofol TCI) compared to those who had DISE with a crude bolus technique. In that prospective trial, the group with standardized TCI sedation had higher postoperative success, presumably because the DISE findings were more accurate and led to better-targeted surgery [48].

The absence of standardized protocols also hampers large-scale data collection. If every center uses its own sedation regimen, pooling data for meta-analyses results in apples-to-oranges comparisons. For example, a meta-analysis might find heterogeneity in the incidence of epiglottis collapse across studies—perhaps explained by the fact that propofol at deeper levels tends to cause a floppier epiglottis than dexmedetomidine sedation would.

Clinical decision-making variability is another consequence. Currently, there are no universal guidelines for how to act on DISE findings. Surgeons primarily rely on their experience to decide which structures to address surgically based on DISE. Some might be aggressive in addressing every level that shows any collapse; others might target only the “main” level. The lack of consensus sedation adds to this subjectivity. As Dijemeni et al. commented, “is the sedation strategy and analysis during DISE objective and systematic?”-implying that standardizing sedation could be a first step to making DISE interpretation more objective [51].

To sum up, lack of standardization remains a significant hurdle in fully realizing DISE’s potential. It affects the reliability (diagnostic variability) and possibly the clinical impact (surgical outcomes). As this field matures, more concerted efforts to define best practices are anticipated. Ultimately, establishing a consistent sedation approach will enable clinicians to place greater confidence in the findings and researchers to more clearly evaluate DISE’s utility in improving patient outcomes.

Beyond optimizing pharmacological protocols, recent technological developments suggest that future diagnostic strategies may partially circumvent the limitations inherent to sedative-induced sleep. Emerging technologies may further challenge the current paradigm of pharmacologically induced sleep endoscopy; notably, Lin et al. [52] recently described a novel anesthesia-free sleep endoscopy system (NasoLens), which enables upper airway visualization during physiological sleep without sedative agents, potentially eliminating sedation-related bias in airway collapsibility assessment.

### 3.7. Special Considerations for Obese OSA Patients

A large OSA subgroup of patients presents obesity as a comorbidity [53]. Given that the prevalence of obesity in patients with OSA has been reported to be as high as 39% among those with any form of OSA and 54% among those with severe OSA [54], and considering the significant and multifaceted pathogenic role of obesity in OSA—particularly at elevated body mass index [53]—it is crucial to select an appropriate pharmacokinetic-pharmacodynamic (PK-PD) model for propofol administration that accounts for the unique pharmacokinetic and pharmacodynamic characteristics of this patient population. The Schnider model, probably the most utilized PK-PD model in propofol TCI sedation for DISE, was primarily created based on data from non-obese patients [32], raising questions about its applicability to obese populations. Obese individuals exhibit distinct pharmacokinetic profiles influenced by factors such as increased fat mass, altered blood flow, and altered volumes of distribution. These differences can impact drug distribution, metabolism, and clearance. Furthermore, administering DISE at the lowest minimally effective effect-site concentration of propofol may reduce the drug’s influence on upper airway dynamics, thereby preserving its function during pharmacologically induced sleep. The increase in airway collapsibility is associated with a dose-dependent inhibition of genioglossus muscle activity by propofol, likely due to a combination of diminished upper airway reflexes and reduced central respiratory output to the airway dilator muscles [55]. Implementing general-purpose pharmacokinetic-pharmacodynamic (PK-PD) models, such as Eleveld, may enhance the precision of sedation management in DISE by providing individualized dosing strategies that optimize safety and efficacy in obese patients.

### 3.8. Diagnostic Variability-Impact on Surgical Decision

Sedation depth significantly affects airway collapsibility and obstruction patterns, with deeper sedation increasing collapse and potentially exaggerating obstruction sites [17,41,45]. The accuracy of drug-induced sleep endoscopy (DISE) directly influences diagnostic reliability, as precise visualization of the upper airway can enhance the identification of obstruction sites and inform targeted treatment strategies. Interobserver agreement is moderate, with better reliability at the oropharynx and velum, but poor agreement at the tongue base [17,46,56,57]. One key question regarding the impact of DISE resides in how to objectively determine surgical treatment decisions during DISE without being influenced by the sedation administration strategy employed [51]. The primary debate centers on the challenge of objectively and systematically confirming that the observed upper airway collapse during DISE is unaffected by the selected sedation administration strategy. Surgical outcomes were improved in patients who received propofol TCI compared to those treated with the bolus technique [19]. At present, there is no established recommendation for the appropriate surgical treatment in cases of varying levels and patterns of obstruction. The decision is largely contingent upon the investigator’s experience and surgical expertise. Moreover, a recent randomized interventional study and data from a systematic review [48,49] revealed that preoperative topo-diagnosis using DISE does not have a significant impact on surgical outcomes for OSA, and primary cases of OSA may benefit from a cost-effective surgical protocol that forgoes DISE and incorporates multilevel interventions completed within a reasonable timeframe.

### 3.9. Diagnostic Variability-Impact of Body Position on DISE Findings and Considerations Regarding Anatomic Airflow Limitation

The standard DISE is usually performed in the supine position for practical reasons, but positional OSA is common, and many patients experience significantly less collapse when not supine [58]. Lateral sleep posture can reduce gravity-related airway narrowing, particularly at the tongue base [58], and performing DISE exclusively supine may therefore overestimate collapse in patients who predominantly sleep laterally. A recent study, performed in 186 OSA patients, showed that changing from supine to lateral position during DISE dramatically reduced airway collapse, especially at the tongue base (in 94.9% of cases, tongue base obstruction resolved in lateral position) [58]. This indicates that supine DISE can overestimate obstruction severity in patients who do not normally sleep supine. Earlier studies have demonstrated position-related differences in collapse patterns [59]. The severity of antero-posterior collapse is less severe during head rotation than in lateral head and trunk position [59]. Whether DISE should be performed in the patient’s habitual sleep position (if non-supine) remains debated, since supine DISE may reveal collapses that would not occur during lateral sleep [60]. Some centers now explore DISE in lateral or semi-lateral positions to identify positional therapy opportunities [58].

It is important to recognize that observing an open airway on DISE does not guarantee normal airflow. In some cases, the site of greatest airflow limitation may not visibly collapse at all, and the structure seen to collapse might not be the primary flow-limiting site of obstruction [61]. In addition, Garcia et al. described scenarios where an upstream segment (e.g., the nasal valve) causes flow limitation, but collapse is only seen downstream at the soft palate or tongue base [62]. Moreover, the hypoglossal nerve stimulation (HNS) studies confirm this airflow–collapse disconnect. For instance, one study found that at low stimulation levels, the airway looked more open but inspiratory flow remained limited until stimulation was increased to abolish flow limitation [63]. During hypoglossal nerve stimulation, investigators observed that residual flow limitation persisted even when the endoscopic view showed an open airway until stimulation was high enough to eliminate the flow plateau [63]. Advanced techniques like placing a nasal pressure catheter or performing simultaneous manometry can identify the true flow-limiting site [61], but these are not routine in clinical DISE because of complexity. Future DISE protocols could benefit from concurrent airflow measurement or pharyngeal pressure monitoring to distinguish primary sites of obstruction from secondary collapses [61], although implementing such monitoring in practice is challenging.

### 3.10. Sedation Considerations for Patients with Comorbidities

Patient comorbidities can significantly alter the pharmacokinetic and pharmacodynamic profiles of sedative agents used during DISE. For instance, chronic obstructive pulmonary disease (COPD) or chronic hypoxemic respiratory failure increases the risk of sedation-induced hypoventilation, which can lead to hypercapnia and hypoxemia. This necessitates cautious dose titration to avert adverse respiratory outcomes. Similarly, patients with severe cardiac impairments, such as dilated cardiomyopathy with arrhythmias, may experience reduced cardiac output that slows drug clearance, intensifying hemodynamic effects and heightening the risk of over-sedation, hypotension, or arrhythmias. Additionally, diabetes mellitus accompanied by end-organ dysfunction often prolongs the metabolism and clearance of these agents, further emphasizing the need for dosage adjustments based on individual health status [64].

Before conducting DISE, thorough assessments of the metabolic and biochemical status of OSA patients are vital. Evaluations of acid–base balance, liver function, renal function, and cardiac status can significantly influence the administration strategy and dosage of hypnotic agents. Identifying any underlying abnormalities in these parameters is essential for devising a safe and effective sedation plan [6].

Importantly, the pharmacokinetic and pharmacodynamic models of propofol or dexmedetomidine were primarily developed from studies involving healthy, young, non-obese populations. As a result, these models may not accurately predict drug behavior in all patients with conditions like OSA, who often present with additional complexities such as advanced age or obesity and multiple comorbidities. Recognizing the limitations of traditional PK-PD models is crucial for the effective management of sedation in patients with comorbid conditions. By integrating these considerations into the clinical management of high-risk patients, we can optimize sedation safety and enhance overall patient outcomes [65].

## 4. Conclusions

In summary, there is significant variability and uncertainty surrounding sedation techniques and protocols for drug-induced sleep endoscopy (DISE) in the evaluation of obstructive sleep apnea (OSA). Despite the integration of DISE as a valuable diagnostic tool, no universally accepted sedation protocol exists. Key considerations include selecting appropriate sedative agents, preventing over-sedation, and carefully titrating to maintain upper airway patency, minimize the risk of collapse, and obtain accurate diagnostic information.

Further research is necessary to standardize sedation protocols and establish guidelines tailored to diverse patient populations, particularly obese patients who present unique pharmacokinetic and pharmacodynamic challenges during DISE. The identification of optimal pharmacokinetic-pharmacodynamic models enabling TCI sedation should be prioritized to enhance the safety and efficacy of propofol administration.

Moreover, the influence of sedation strategies on surgical decision-making remains a critical area for investigation. As surgical outcomes continue to be influenced by individual clinicians’ experience and preferences, objective decision-making criteria based on DISE findings require further development. This may reduce unnecessary surgical interventions and ensure patients receive the most effective care.

Given the conflicting literature on DISE’s role in surgical treatment decisions, a more concerted effort to establish robust protocols and data-driven recommendations is essential. This effort will help refine the evaluation and management of patients with OSA, ultimately improving patient outcomes and safety in clinical practice.

## Figures and Tables

**Table 1:** Comparison of key sedative agents used in DISE.

Characteristic	Propofol	Dexmedetomidine	Midazolam
**Onset of** **sedation**	Rapid—~1 min to effect; ideal for quick titration.	Slower—~5–10 min infusion for full effect; titrate gradually.	Moderate—~2–3 min to initial effect (IV); slower peak.
**Recovery time**	Short—quick offset when infusion stopped (metabolized rapidly).	Longer—sedation may linger; patients need longer monitoring.	Intermediate to long–sedative effect can persist (active metabolites).
**Airway** **muscle** **effects**	Marked relaxation; dose-dependent reduction in pharyngeal muscle tone and reflexes. High doses can induce collapsibility beyond natural sleep levels.	Mild relaxation; sedation mimics natural sleep stages. Preserves greater muscle tone, resulting in less airway collapse at equivalent sedation levels.	Moderate relaxation; similar propensity for airway obstruction as propofol at moderate sedation. It can significantly depress airway reflexes with increasing dose.
**Respiratory** **effects**	Respiratory depressant—risk of apnea at deeper levels. Requires airway monitoring/support.	Minimal respiratory depression—maintains spontaneous breathing; lower risk of apnea.	Respiratory depressant—can cause hypoventilation especially if combined with opioids. Unpredictable at higher doses.
**Hemodyn** **amic** **profile**	Reduces systemic vascular resistance—may cause hypotension; mild myocardial depression. Heart rate can increase or decrease (varies).	Stable with bradycardia—can cause bradycardia and modest BP reductions (central sympatholysis) Generally preserves hemodynamics well, few stress responses.	Typically mild BP reduction; can cause hypotension especially in frail patients. Reduced control can lead to sudden drops if oversedated.
**Diagnostic reliability**	Well-studied; widely used in DISE. If titrated to moderate sedation, provides collapse patterns similar to natural sleep. Over-sedation can cause false collapse.	Sedation state very close to physiologic sleep; tends to show true collapse sites without excessive relaxation. Might under-represent the most severe collapses (no REM atonia).	Can reproduce OSA collapse patterns in Stage N2 sleep. Agreement with propofol observed. Depth must be controlled to avoid missing events or over-relaxing airway.
**Advantages for DISE**	Fast on/off, easily titrated (especially with TCI). Extensive experience and evidence base. Short duration suits outpatient DISE.	Natural sleep-like sedation, minimal interference with breathing, analgesic properties. Good for patients with respiratory risk (severe OSA, obesity hypoventilation). Has reversal agent (atipamezole) if needed.	Widely available, familiar. Provides amnesia. Has an antagonist (flumazenil) for reversal. Can be given in settings without infusion pumps.
**Limitations for DISE**	Narrow margin between adequate and excessive sedation—risk of airway collapse from drug effect. Requires anesthesiology support. No reversal agent.	Slow onset and longer recovery—less efficient workflow. Requires an infusion pump. Cost can be higher. Bradycardia in some patients. Limited data on long-term outcomes.	Harder to fine-tune level—risk of over- or under-sedation. Respiratory depression, especially with co-sedatives. Longer hangover sedation. Not ideal for precise depth control (TCI is not widely available).

Data summarized from previously published clinical and physiological studies: [Carrasco Llatas et al., Eur Arch ORL, 2014, Ref. [21]]—propofol vs. midazolam, [Eastwood et al., Anesthesiology, 2005—airway collapsibility vs. propofol dose, Ref. [4]], [Shteamer & Dedhia, Laryngoscope, 2017. Ref. [13]]—neuropharmacology review, [Yoon et al., Laryngoscope, 2016. Ref. [14]]—propofol vs. dexmedetomidine, [Viana et al., Laryngoscope, 2019. Ref. [26]]—sedative agents and DISE findings, [Heiser et al., Sleep Breath, 2017, Ref. [18]]—sedation depth relevance.

## Data Availability

No new data were created or analyzed in this study. Data sharing is not applicable to this article.

## Abbreviations

The following abbreviations are used in this manuscript:

DISE	Drug-Induced Sleep Endoscopy
OSA	Obstructive Sleep Apnea
TCI	Target-Controlled Infusion
PK–PD	Pharmacokinetic–Pharmacodynamic
BIS	Bispectral Index

## References

[1] Chang J.L., Goldberg A.N., Alt J.A., Mohammed A., Ashbrook L., Auckley D., Ayappa I., Bakhtiar H., Barrera J.E., Bartley B.L. (2023). International Consensus Statement on Obstructive Sleep Apnea. Int. Forum Allergy Rhinol..

[2] Chaudhry R.A., Zarmer L., West K., Chung F. (2024). Obstructive Sleep Apnea and Risk of Postoperative Complications after Non-Cardiac Surgery. J. Clin. Med..

[3] Luzzi V., Mazur M., Guaragna M., Di Carlo G., Cotticelli L., Magliulo G., Marasca B., Pirro V., Di Giorgio G., Ndokaj A. (2022). Correlations of Obstructive Sleep Apnea Syndrome and Daytime Sleepiness with the Risk of Car Accidents in Adult Working Population: A Systematic Review and Meta-Analysis with a Gender-Based Approach. J. Clin. Med..

[4] Eastwood P.R., Platt P.R., Shepherd K., Maddison K., Hillman D.R. (2005). Collapsibility of the upper airway at different concentrations of propofol anesthesia. Anesthesiology.

[5] De Vito A., Carrasco Llatas M., Vanni A., Bosi M., Braghiroli A., Campanini A., de Vries N., Hamans E., Hohenhorst W., Kotecha B.T. (2014). European position paper on drug-induced sedation endoscopy (DISE). Sleep Breath..

[6] De Vito A., Carrasco Llatas M., Ravesloot M.J., Kotecha B., de Vries N., Hamans E., Maurer J., Bosi M., Blumen M., Heiser C. (2018). European position paper on drug-induced sleep endoscopy: 2017 Update. Clin. Otolaryngol..

[7] Olszewska E., De Vito A., Heiser C., Vanderveken O., O’Connor-Reina C., Baptista P., Kotecha B., Vicini C. (2024). Consensus Statements among European Sleep Surgery Experts on Snoring and Obstructive Sleep Apnea: Part 3 Palatal Surgery, Outcomes and Follow-Up, Complications, and Post-Operative Management. J. Clin. Med..

[8] Vroegop A.V., Vanderveken O.M., Verbraecken J.A. (2020). Drug-Induced Sleep Endoscopy: Evaluation of a Selection Tool for Treatment Modalities for Obstructive Sleep Apnea. Respiration.

[9] Green K.K., Kent D.T., D’Agostino M.A., Hoff P.T., Lin H.S., Soose R.J., Boyd Gillespie M., Yaremchuk K.L., Carrasco-Llatas M., Tucker Woodson B. (2019). Drug-Induced Sleep Endoscopy and Surgical Outcomes: A Multicenter Cohort Study. Laryngoscope.

[10] Bastier P.L., Gallet de Santerre O., Bartier S., De Jong A., Trzepizur W., Nouette-Gaulain K., Bironneau V., Blumen M., Chabolle F., de Bonnecaze G. (2022). Guidelines of the French Society of ENT (SFORL): Drug-induced sleep endoscopy in adult obstructive sleep apnea syndrome. Eur. Ann. Otorhinolaryngol. Head Neck Dis..

[11] Qi Y., Zhao Y., Yan Y., Wu D. (2024). Surgical failure guided by DISE in patients with obstructive sleep apnea: A systematic review and meta-analysis. Eur. Arch. Otorhinolaryngol..

[12] Schwartz D., Schall C., Harders M., Tollinche L., Tanios M., Alter J., Weidenbecher M. (2024). Anesthesia for Drug Induced Sleep Endoscopy (DISE). Curr. Anesthesiol. Rep..

[13] Shteamer J.W., Dedhia R.C. (2017). Sedative choice in drug-induced sleep endoscopy: A neuropharmacology-based review. Laryngoscope.

[14] Yoon B.W., Hong J.M., Hong S.L., Koo S.K., Roh H.J., Cho K.S. (2016). A comparison of dexmedetomidine versus propofol during drug-induced sleep endoscopy in sleep apnea patients. Laryngoscope.

[15] Wan C.C.J., Leung Y.Y. (2025). Current Concepts of the Applications and Treatment Implications of Drug-Induced Sleep Endoscopy for the Management of Obstructive Sleep Apnoea. Diagnostics.

[16] Capasso R., Rosa T., Tsou D.Y., Nekhendzy V., Drover D., Collins J., Zaghi S., Camacho M. (2016). Variable Findings for Drug-Induced Sleep Endoscopy in Obstructive Sleep Apnea with Propofol versus Dexmedetomidine. Otolaryngol. Head Neck Surg..

[17] Traxdorf M., Tschaikowsky K., Scherl C., Bauer J., Iro H., Angerer F. (2016). Drug-Induced Sleep Endoscopy (DISE) with Target Controlled Infusion (TCI) and Bispectral Analysis in Obstructive Sleep Apnea. J. Vis. Exp..

[18] Heiser C., Fthenakis P., Hapfelmeier A., Berger S., Hofauer B., Hohenhorst W., Kochs E.F., Wagner K.J., Edenharter G.M. (2017). Drug-induced sleep endoscopy with target-controlled infusion using propofol and monitored depth of sedation to determine treatment strategies in obstructive sleep apnea. Sleep Breath..

[19] De Vito A., Agnoletti V., Zani G., Corso R.M., D’aGostino G., Firinu E., Marchi C., Hsu Y.-S., Maitan S., Vicini C. (2017). The importance of drug-induced sedation endoscopy (D.I.S.E.) techniques in surgical decision making: Conventional versus target controlled infusion techniques—A prospective randomized controlled study and a retrospective surgical outcomes analysis. Eur. Arch. Oto-Rhino-Laryngol..

[20] Padiyara T.V., Bansal S., Jain D., Arora S., Gandhi K. (2020). Dexmedetomidine versus propofol at different sedation depths during drug-induced sleep endoscopy: A randomized trial. Laryngoscope.

[21] Carrasco Llatas M., Agostini Porras G., Cuesta González M.T., Rodrigo Sanbartolomé A., Giner Bayarri P., Gómez-Pajares F., Dalmau Galofre J. (2014). Drug-induced sleep endoscopy: A two drug comparison and simultaneous polysomnography. Eur. Arch. Otorhinolaryngol..

[22] Carrasco-Llatas M., Matarredona-Quiles S., De Vito A., Chong K.B., Vicini C. (2019). Drug-induced sleep endoscopy: Technique, indications, tips and pitfalls. Healthcare.

[23] Han L., Drover D.R., Chen M.C., Saxena A.R., Eagleman S.L., Nekhendzy V., Pritchard A., Capasso R. (2023). EEG response of dexmedetomidine during drug induced sleep endoscopy. Front. Neurosci..

[24] Li C., Fan H., Duan Y., Wang D., Lin Y., Xin W., Ma R., Wen W., Wu Y. (2025). Effects of dexmedetomidine and propofol on the key endotypes of OSA: A randomized, single-blind, placebo-controlled, crossover trial. Sleep Med..

[25] Zhang J., Zhang Y., Fang X., Weng L., Zhu S., Luo N., Huang D., Guo Q., Huang C. (2023). Comparison of Remimazolam and Propofol for Drug-Induced Sleep Endoscopy: A Randomized Clinical Trial. Otolaryngol. Head Neck Surg..

[26] Viana A., Zhao C., Rosa T., Couto A., Neves D.D., Araújo-Melo M.H., Capasso R. (2019). The Effect of Sedating Agents on Drug-Induced Sleep Endoscopy Findings. Laryngoscope.

[27] Tănase N.V., Hainăroșie R., Brîndușe L.A., Cobilinschi C., Dutu M., Corneci D., Zainea V. (2024). Study of Two Sedative Protocols for Drug-Induced Sleep Endoscopy: Propofol versus Propofol-Remifentanil Combination, Delivered in Target-Controlled Infusion Mode. Medicina.

[28] Cho J.S., Soh S., Kim E.J., Cho Hju Shin S., Kim H.J., Koo B.N. (2015). Comparison of three sedation regimens for drug-induced sleep endoscopy. Sleep Breath..

[29] Kim Y., Park H., Shin J., Choi J.H., Park S.W., Kang H.Y. (2018). Effect of remifentanil during drug-induced sleep endoscopy in patients with obstructive sleep apnea. Sleep Breath..

[30] Šafránková P., Bruthans J. (2025). Target-Controlled Infusion of Propofol: A Systematic Review of Recent Results. J. Med. Syst..

[31] Marsh B., White M., Morton N., Kenny G.N. (1991). Pharmacokinetic model driven infusion of propofol in children. Br. J. Anaesth..

[32] Schnider T.W., Minto C.F., Gambus P.L., Andresen C., Goodale D.B., Shafer S.L., Youngs E.J. (1998). The influence of method of administration and covariates on the pharmacoki-netics of propofol in adult volunteers. Anesthesiology.

[33] Eleveld D.J., Colin P., Absalom A.R., Struys M.M.R.F. (2018). Pharmacokinetic-pharmacodynamic model for propofol for broad application in anaesthesia and sedation. Br. J. Anaesth..

[34] Vellinga R., Eleveld D.J., Struys M.M.R.F., van den Berg J.P. (2023). General purpose models for intravenous anesthetics, the next generation for target-controlled infusion and total intravenous anesthesia?. Curr. Opin. Anaesthesiol..

[35] Tănase N.-V., Hainăroșie R., Brîndușe L.-A., Corneci D., Voiosu C., Rusescu A., Cobilinschi C., Stanciu Găvan C., Zainea V. (2025). A Clinical Comparative Study of Schnider and Eleveld Pharmacokinetic–Pharmacodynamic Models for Propofol Target-Controlled Infusion Sedation in Drug-Induced Sleep Endoscopy. Biomedicines.

[36] Elkalla R.S., El Mourad M.B. (2020). Respiratory and hemodynamic effects of three different sedative regimens for drug induced sleep endoscopy in sleep apnea patients. A prospective randomized study. Minerva Anestesiol..

[37] Yongping Z., Xinyi L., Aming S., Qiang X., Tianqi Z., Mengmeng S., Xiong C., Xuemin S. (2022). The safety and efficacy of esketamine in comparison to dexmedetomidine during drug-induced sleep endoscopy in children with obstructive sleep apnea hypopnea syndrome: A randomized, controlled and prospective clinical trial. Front. Pharmacol..

[38] Chen Y.T., Sun C.K., Wu K.Y., Chang Y.J., Chiang M.H., Chen I.W., Liao S.W., Hung K.C. (2021). The Use of Propofol versus Dexmedetomidine for Patients Receiving Drug-Induced Sleep Endoscopy: A Meta-Analysis of Randomized Controlled Trials. J. Clin. Med..

[39] Kandil A., Subramanyam R., Hossain M.M., Ishman S., Shott S., Tewari A., Mahmoud M. (2016). Comparison of the combination of dexmedetomidine and ketamine to propofol or propofol/sevoflurane for drug-induced sleep endoscopy in children. Pediatr. Anesth..

[40] Ehsan Z., Mahmoud M., Shott S.R., Amin R.S., Ishman S.L. (2016). The effects of anesthesia and opioids on the upper airway: A systematic review. Laryngoscope.

[41] Lo Y.L., Ni Y.L., Wang T.Y., Lin T.Y., Li H.Y., White D.P., Lin J.R., Kuo H.P. (2015). Bispectral Index in Evaluating Effects of Sedation Depth on Drug-Induced Sleep Endoscopy. J. Clin. Sleep Med..

[42] Nieuwenhuijs D., Coleman E.L., Douglas N.J., Drummond G.B., Dahan A. (2002). Bispectral index values and spectral edge frequency at different stages of physiologic sleep. Anesth. Analg..

[43] Stierer T.L., Ishman S.L. (2015). Bispectral Index in Evaluating Effects of Sedation Depth on Drug-Induced Sleep Endoscopy: DISE or No Dice. J. Clin. Sleep Med..

[44] Rasulo F.A., Hopkins P., Lobo F.A., Pandin P., Matta B., Carozzi C., Romagnoli S., Absalom A., Badenes R., Bleck T. (2023). Processed Electroencephalogram-Based Monitoring to Guide Sedation in Critically Ill Adult Patients: Recommendations from an International Expert Panel-Based Consensus. Neurocrit. Care.

[45] Hight D., Kreuzer M., Ugen G., Schuller P., Stüber F., Sleigh J., Kaiser H.A. (2023). Five commercial ‘depth of anaesthesia’ monitors provide discordant clinical recommendations in response to identical emergence-like EEG signals. Br. J. Anaesth..

[46] Bartier S., Blumen M., Chabolle F. (2020). Is image interpretation in drug-induced sleep endoscopy that reliable?. Sleep Breath..

[47] Vlad A.M., Stefanescu C.D., Stefan I., Zainea V., Hainarosie R. (2023). Comparative Efficacy of Velopharyngeal Surgery Techniques for Obstructive Sleep Apnea: A Systematic Review. Medicina.

[48] Di Bari M., Colombo G., Giombi F., Leone F., Bianchi A., Colombo S., Salamanca F., Cerasuolo M. (2024). The effect of drug-induced sleep endoscopy on surgical outcomes for obstructive sleep apnea: A systematic review. Sleep Breath..

[49] Askar S.M., Khazbak A.O., Mobasher M.A., Abd Al Badea A.M., Abu Sharkh A.A., Awad A.M. (2023). Role of DISE in the surgical outcome for patients with obstructive sleep apnea. Am. J. Otolaryngol..

[50] Lisan Q., Baudouin R., Lechien J.R., Hans S., Blumen M. (2023). Is drug-induced sleep endoscopy associated with better outcomes after soft tissue surgery for sleep apnea? A systematic review and meta-analysis. Clin. Otolaryngol..

[51] Dijemeni E., D’Amone G. (2018). Is sedation administration strategy and analysis during drug-induced sedation endoscopy objective and systematic?. Sleep Breath..

[52] Lin Y.T., Shih C.W., Chen N., Lu H.T., Chu W.C., Chen K.C. (2025). Eliminating the Need for Anesthesia in Sleep Endoscopy: A Comparative Study of Traditional Nasopharyngoscope Design Versus NasoLens. Bioengineering.

[53] Messineo L., Bakker J.P., Cronin J., Yee J., White D.P. (2024). Obstructive sleep apnea and obesity: A review of epidemiology, pathophysiology and the effect of weight-loss treatments. Sleep Med. Rev..

[54] Esmaeili N., Gell L., Taranto-Montemurro L., Messineo L., Imler T., Sands S.A., Yee J., Cronin J., Wellman A., White D.P. (2024). Prevalence of obesity in obstructive sleep apnea within a large community-based cohort of middle-aged/older adults. Sleep.

[55] Simons J.C., Pierce E., Diaz-Gil D., Malviya S.A., Meyer M.J., Timm F.P., Stokholm J.B., Rosow C.E., Kacmarek R.M., Eikermann M. (2016). Effects of Depth of Propofol and Sevoflurane Anesthesia on Upper Airway Collapsibility, Respiratory Genioglossus Activation, and Breathing in Healthy Volunteers. Anesthesiology.

[56] Mitsikas D., Jakob B., Janjic V., Hasler C., Tschopp S. (2024). Interrater reliability of different scoring systems for drug-induced sleep endoscopy. Sleep Breath..

[57] Friedman N.R., Parikh S.R., Ishman S.L., Ruiz A.G., El-Hakim H., Ulualp S.O., Wootten C.T., Koltai P.J., Chan D.K. (2017). The current state of pediatric drug-induced sleep endoscopy. Laryngoscope.

[58] Mladoňová M., Fedorová K., Jor O., Slonková J., Kondé A., Komínek P., Matoušek P. (2025). The role of positional changes in optimizing OSA treatment: Evidence from DISE. Eur. Arch. Otorhinolaryngol..

[59] Safiruddin F., Koutsourelakis I., de Vries N. (2015). Upper airway collapse during drug induced sleep endoscopy: Head rotation in supine position compared with lateral head and trunk position. Eur. Arch. Otorhinolaryngol..

[60] Landry S.A., Beatty C., Thomson L.D.J., Wong A.M., Edwards B.A., Hamilton G.S., Joosten S.A. (2023). A review of supine position related obstructive sleep apnea: Classification, epidemiology, pathogenesis and treatment. Sleep Med. Rev..

[61] Harkins T., Tangutur A., Keenan B.T., Seay E.G., Thuler E., Dedhia R.C., Schwartz A.R. (2024). Pharyngeal Manometry and Upper Airway Collapse During Drug-Induced Sleep Endoscopy. JAMA Otolaryngol. Head Neck Surg..

[62] Garcia G.J.M., Woodson B.T. (2020). The collapsing anatomical structure is not always the primary site of flow limitation in obstructive sleep apnea. J. Clin. Sleep Med..

[63] Schwartz A.R., Barnes M., Hillman D., Malhotra A., Kezirian E., Smith P.L., Hoegh T., Parrish D., Eastwood P.R. (2012). Acute upper airway responses to hypoglossal nerve stimulation during sleep in obstructive sleep apnea. Am. J. Respir. Crit. Care Med..

[64] Fox C., Liu H., Yarborough M., Fox M.E., Kaye A.D. (2012). High-risk patients: Sedation considerations in coexisting disease. Moderate and Deep Sedation in Clinical Practice.

[65] Masui K., Absalom A.R., Mason K.P. (2017). How to select a PK/PD model. Total Intravenous Anesthesia and Target Controlled Infusions—A Comprehensive Global Anthology.

